# The Bottle of Medicine Habit

**Published:** 1923-05

**Authors:** 


### The Bottle of Medicine Habit.

Sir,—I rejoiced to see the letter on this subject from "A Consultant" in your April number. Some people—many of them—are fond of drams of alcohol; others—nearly as many—are equally fond of drams of drugs. One batch is almost as bad as the other, and every general practitioner knows how happy "a bottle of medicine" makes a certain type of patient. The vogue of patent medicines is ever increasing; so is the harm they do. If the sale of these things were more strictly controlled the "bottle of medicine habit" would receive a distinct check; but the revenue benefits largely, and as long as that is the case I fear that particular evil will flourish.

